# Case report: co-inheritance of familial lecithin-cholesterol acyltransferase deficiency and α^0^-Thalassemia

**DOI:** 10.3389/fgene.2026.1806855

**Published:** 2026-04-17

**Authors:** Yinbing Zhu, Chunya Liu, Wenxun Tang, Yi Jiang, Lingling Mao, Min Huang

**Affiliations:** Department of Nephrology, The Quzhou Affiliated Hospital of Wenzhou Medical University, Quzhou People’s Hospital, Quzhou, Zhejiang, China

**Keywords:** co-inheritance, consanguineous marriage, LCAT deficiency, low high-density lipoprotein cholesterol, nephrotic syndrome, α^0^-thalassemia

## Abstract

**Background:**

Familial lecithin-cholesterol acyltransferase (*LCAT*) deficiency and α^0^-thalassemia are rare autosomal recessive disorders. Although both disease-causing genes reside on chromosome 16, their physical distance typically results in independent inheritance in non-consanguineous populations. Co-inheritance of both conditions has not been previously reported.

**Case presentation:**

A 50-year-old Chinese man with childhood-onset corneal opacity and long-standing anemia presented with 2 months of progressive lower limb edema. Laboratory evaluation revealed nephrotic syndrome and markedly reduced high-density lipoprotein cholesterol (HDL-C). Renal biopsy showed characteristic glomerular lipid deposition, confirming *LCAT* deficiency. Genetic testing identified a homozygous *LCAT* mutation (c.355G>C, p.Gly119Arg), with both parents confirmed as heterozygous carriers. The patient had severe microcytic hypochromic anemia that did not fully align with the mild hemolytic anemia typical of *LCAT* deficiency. Given parental consanguinity, expanded genetic testing revealed co-inheritance of α^0^-thalassemia (*HBA*: -SEA/αα), explaining the hematological phenotype.

**Outcome:**

No specific treatment exists for *LCAT* deficiency. Symptomatic management with angiotensin-converting enzyme inhibitors and diuretics improved edema. The α^0^-thalassemia trait is asymptomatic and requires no intervention; its diagnosis avoided unnecessary iron therapy and the associated risk of iron overload. Long-term follow-up will focus on renal function, proteinuria, lipid profile, and ocular findings. Genetic counseling will also be provided to the patient and their family.

**Conclusion:**

To our knowledge, this is the first reported case of co-inherited *LCAT* deficiency and α^0^-thalassemia confirmed by both renal pathology and comprehensive genetic testing. The consanguineous background suggests possible co-transmission of distant recessive variants on the same chromosome. This case highlights the importance of considering coexisting genetic disorders in patients with consanguinity or unexplained multisystem involvement.

## 1 Introduction

Familial lecithin-cholesterol acyltransferase (*LCAT*) deficiency is a rare autosomal recessive genetic disorder, with an incidence of less than one in 1,000,000. This condition results in abnormal lipid metabolism, characterized by a significant reduction in high-density lipoprotein cholesterol (HDL-C). The disorder also leads to corneal opacity, hemolytic (often normocytic) anemia, and progressive renal damage (Funke et al., 1993; Calabresi et al., 2005; Lamiquiz-Moneo et al., 2019; Pavanello and Calabresi, 2020). Due to the heterogeneous clinical manifestations, some patients may first present with edema, proteinuria, or hematuria, while others may exhibit atypical lipid profiles, which poses a diagnostic challenge. Histopathological findings in the kidneys and genetic testing play a crucial role in confirming the diagnosis.

α^0^-thalassemia is an autosomal recessive disorder, commonly caused by deletions or mutations in the *HBA* (α-globin) gene. The main clinical manifestation is microcytic hypochromic anemia (Santamarina-Fojo et al., 2000; Ozkok et al., 2017; Kattamis et al., 2022; Miyata et al., 2025). When combined with other genetic disorders, such as *LCAT* deficiency, the hematological phenotype may be atypical. This overlap can obscure the clinical picture of the primary disease and delay diagnosis, as well as hinder the initiation of appropriate treatment.

Although both *LCAT* deficiency and α^0^-thalassemia are caused by genes located on opposite arms of chromosome 16 [*LCAT* at 16q22.1, the *HBA* (α-globin) gene at 16p13.3] and are typically inherited independently in non-consanguineous populations, consanguineous marriage increases the likelihood of homozygosity for multiple recessive alleles, potentially leading to their co-inheritance (Jaouad et al., 2009; de Serpa Brandão et al., 2022; Khayat et al., 2024). To assess the novelty of this co-inheritance, we conducted a systematic literature search in PubMed, Web of Science, and CNKI databases (up to January 2026). While co-inheritance of β-thalassemia with *LCAT* deficiency has been reported (Utermann et al., 1981), no previous case of α^0^-thalassemia co-inherited with *LCAT* deficiency was identified. Thus, to our knowledge, the present case represents the first documented instance confirmed by renal pathology and comprehensive genetic testing. Herein, we report a patient who presented with bilateral lower-limb edema, nephrotic syndrome, and markedly low HDL-C, in whom combined renal biopsy and genetic testing established the diagnosis of *LCAT* deficiency and α^0^-thalassemia.

## 2 Case presentation

The patient is a 50-year-old Chinese male who was admitted with progressive edema in both lower limbs for the past 2 months. He had a history of bilateral corneal opacity since childhood and long-standing mild anemia-related symptoms, although no systematic diagnostic work-up or regular follow-up had been done. Over the last 2 months, his edema worsened, accompanied by foamy urine, fatigue, and reduced activity tolerance. There were no symptoms of fever, rash, joint pain, gross hematuria, or urinary tract irritation. The patient denied a history of diabetes, hypertension, or chronic liver disease, and reported no long-term use of nephrotoxic medications or unknown Chinese herbal products. His family history indicated consanguinity between his parents. He has one sister who is reportedly healthy with no history of anemia, renal disease, or visual disturbances; however, she declined genetic testing.

Initial examination at another hospital revealed serum albumin of 29.1 g/L and urinary protein of 3+. Given the possibility of nephrotic syndrome, he was referred to our hospital for further evaluation. Physical examination on admission revealed the following: height 185 cm, weight 82.7 kg, BMI 24.2 kg/m^2^, and moderate pitting edema of both lower limbs. Laboratory investigations revealed features characteristic of nephrotic syndrome: serum albumin was 26.2 g/L, 24-h urinary protein excretion was 11.82 g/d, and the urinary albumin-to-creatinine ratio was 5949.18 μg/mg. Renal function was slightly impaired, with a serum creatinine of 115.5 μmol/L (eGFR 63.25 mL/min/1.73 m^2^), while blood urea nitrogen was 5 mmol/L. The blood lipid profile was abnormal: triglycerides 1.73 mmol/L, total cholesterol 3.85 mmol/L, HDL-C 0.43 mmol/L, and low-density lipoprotein cholesterol 1.69 mmol/L. This pattern was characterized by a marked reduction in HDL-C. Autoantibodies (including antinuclear antibodies, anti-neutrophil cytoplasmic antibodies), immunoglobulins, light chains, and anti-phospholipase A2 receptor antibodies were all normal.

**FIGURE 1:**
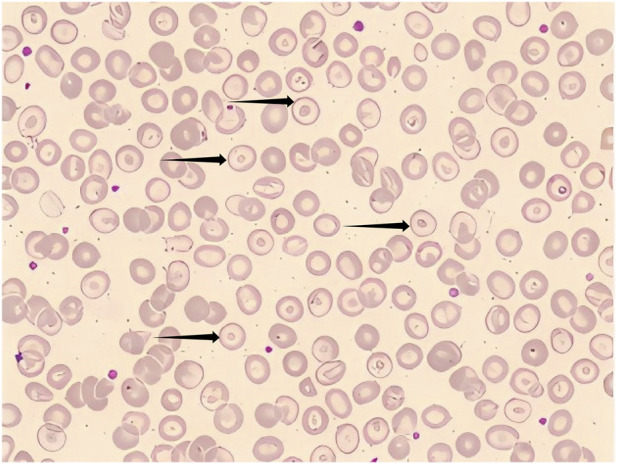
Peripheral blood smear showing microcytic hypochromic anemia with numerous target cells (black arrows). Wright-Giemsa stain, ×1000.

A complete blood count revealed anemia with microcytic hypochromic characteristics: Hb 94 g/L, MCV 68.6 fL, MCH 21.4 pg, MCHC 312 g/L, and a reticulocyte count of 3.22%. Peripheral blood smears confirmed these findings (Figure 1). To further investigate the anemia, iron metabolism tests were performed, showing serum iron of 7.3 μmol/L, total iron-binding capacity of 34.97 μmol/L, transferrin saturation of 0.21%, ferritin 196.41 ng/mL, and transferrin 1.4 g/L. The normal ferritin level ruled out iron deficiency, as low ferritin is a hallmark of depleted iron stores. Low transferrin saturation with normal ferritin is more consistent with anemia of chronic disease or inflammation. Coombs test and flow cytometry for paroxysmal nocturnal hemoglobinuria (PNH) were negative, excluding autoimmune hemolysis and PNH. Erythrocyte osmotic fragility was increased: hemolysis began at 3.6 g/L NaCl (control 4.0–4.5) and was complete at 2.6 g/L NaCl (control 3.0–3.5), suggesting a membrane abnormality or hemoglobinopathy.

Given the clinical findings, the anemia phenotype did not fully align with the anemia typically seen in *LCAT* deficiency. This suggests the possibility of a combined genetic anemia or another underlying cause.

**FIGURE 2:**
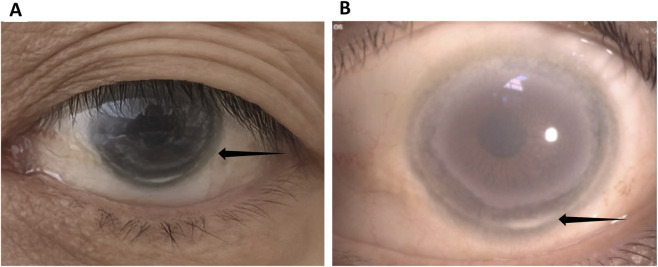
Corneal findings in *LCAT* deficiency. **(A)** Cloudy cornea with fisheye-like appearance. **(B)** Slit-lamp examination showing annular corneal opacity (black arrows).

**FIGURE 3:**
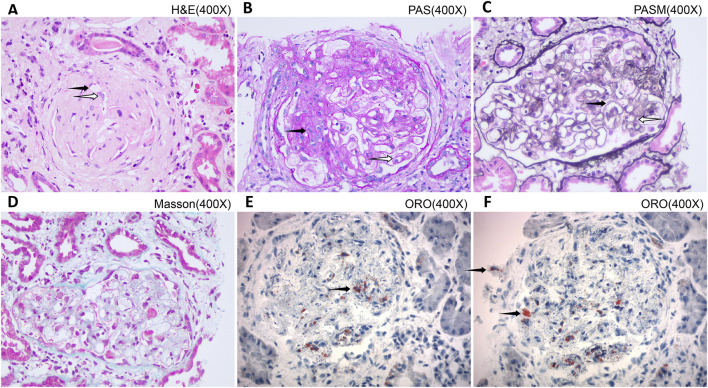
Light microscopy findings on renal biopsy. **(A)** H&E staining: dilated capillary loops with vacuolar thrombus-like material (black arrows) and foamy endothelial cells (white arrows). **(B)** PAS staining: mesangial proliferation (black arrows) and basement membrane thickening (white arrows). **(C)** PASM staining: vacuolar areas in basement membrane (white arrows) with double-track sign (black arrows). **(D)** Masson’s stain: negative for immune deposits. **(E,F)** Oil red O stain: positive lipid droplets in glomeruli and tubular cells (black arrows). ×400.

**FIGURE 4:**
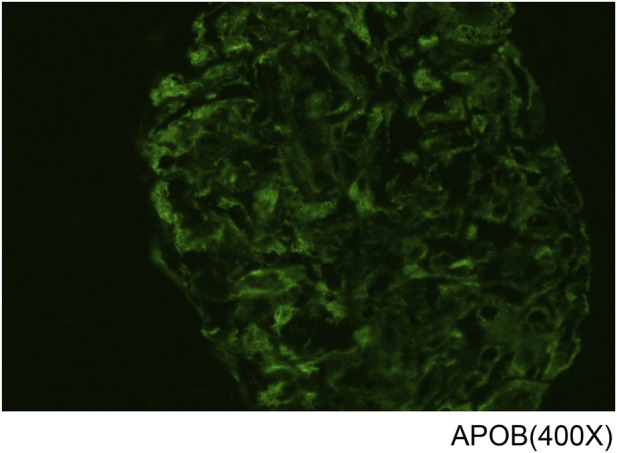
Immunofluorescence staining showing ApoB deposition in glomerular capillary lumina and mesangium. IgA, IgG, IgM, C3, C4, C1q, fibrinogen, κ and λ light chains were negative (not shown). ×400.

**FIGURE 5:**
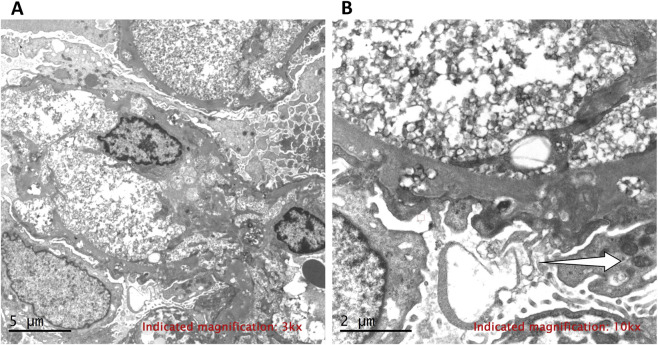
Electron microscopy showing lipid deposits. **(A)** Lipid accumulation in basement membrane, mesangium, and endothelium with foot process fusion. ×3,000. **(B)** Vacuoles with osmiophilic lamellar structures (white arrows). ×10,000.

Given the patient’s history of corneal opacity since childhood (Figure 2), lipid abnormalities with a marked reduction in HDL-C, and nephrotic syndrome, hereditary lipoprotein metabolic disorders, particularly *LCAT* deficiency-related nephropathy, were strongly suspected. To clarify the pathological nature of the renal involvement, a renal biopsy was performed. Histopathological examination revealed glomerular lipid deposition, confirming the diagnosis of *LCAT* deficiency-related renal damage (Figures 3–5).

**FIGURE 6:**
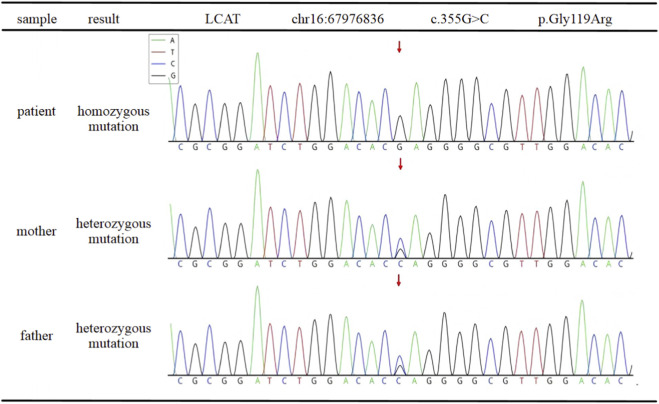
LCAT gene analysis and family verification: The patient was homozygous for the c.355G>C variant. Both parents were asymptomatic carriers of the heterozygous c.355G>C variant.

Based on the pathological findings, targeted next-generation sequencing of hereditary kidney disease genes was performed using a custom panel. Genomic DNA was extracted from peripheral blood samples of the patient and his parents. Illumina sequencing (150 bp paired-end reads) achieved a mean depth of 385×, with 98.7% of target regions covered at ≥30×. Reads were aligned to the GRCh37/hg19 reference genome, and variant calling followed standard bioinformatics pipelines. Population frequencies were obtained from public databases (1000 Genomes, gnomAD, ExAC), and variants were interpreted according to ACMG guidelines. A homozygous mutation in the *LCAT* gene (c.355G>C, p.Gly119Arg) was identified. Sanger sequencing validated this variant in the proband and both parents, confirming heterozygous carrier status in the parents and autosomal recessive inheritance (Figure 6). Combined with the typical clinical manifestations (corneal opacity, significant decrease of HDL-C, nephrotic syndrome) and pathological features of renal tissue, the patient was finally diagnosed as familial *LCAT* deficiency.

To further assess the potential impact of this novel variant, we performed *in silico* pathogenicity predictions using three online tools. PolyPhen-2 scored 0.998 (probably damaging), SIFT scored 0.00 (deleterious), and CADD Phred score was 28.5 (clinically significant, >20) (Table 1). These computational predictions consistently support the pathogenic nature of the c.355G>C (p.Gly119Arg) variant.

**TABLE 1:** In silico pathogenicity predictions for the *LCAT* c.355G>C (p.Gly119Arg) variant.

Prediction tool	Website	Score	Threshold	Prediction
PolyPhen-2	http://genetics.bwh.harvard.edu/pph2/	0.998	>0.9 = probably damaging	Probably damaging
SIFT	https://sift.bii.a-star.edu.sg/	0.00	≤0.05 = deleterious	Deleterious
CADD	https://cadd.gs.washington.edu/	28.5	>20 = clinically significant	Clinically significant

**FIGURE 7:**
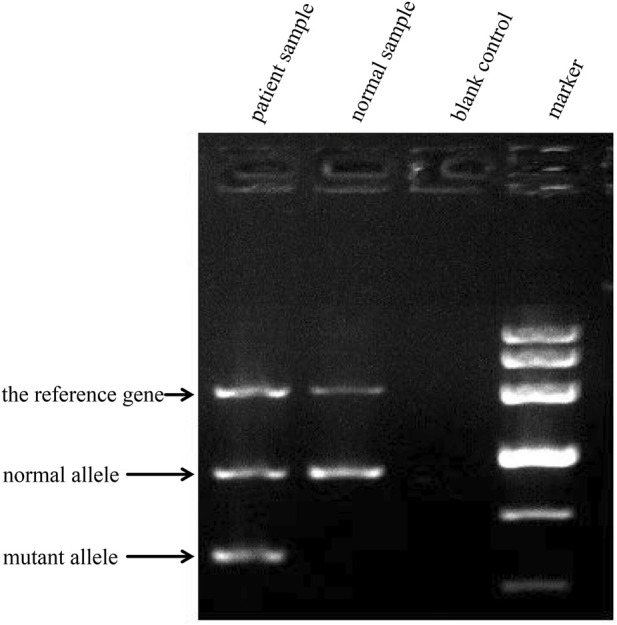
Results of agarose gel electrophoresis of thalassemia-related genes in α^0^-thalassemia.

Notably, the patient’s microcytic hypochromic anemia was not fully explained by the mild hemolytic anemia typical of *LCAT* deficiency. Given the parental consanguinity, we expanded genetic testing to investigate inherited causes. Thalassemia genotyping using CNVplex® and SNPScan® technologies (multiplex PCR, probe hybridization, capillary electrophoresis) targeted 75 common pathogenic variants: 16 α-globin deletions, four homologous recombinations, 10 α-globin point mutations, 8 β-globin deletions, and 37 β-globin point mutations (Table 2). Gap-PCR confirmed the*--SEA* deletion (Figure 7). The genotype (*HBA*: *-SEA/αα*) established α^0^ -thalassemia trait, definitively explaining the anemia. Therefore, the clinical combination of nephrotic syndrome, significantly low HDL-C, and corneal opacity was attributed to *LCAT* deficiency, while the atypical anemia phenotype was linked to α^0^-thalassemia, forming a comprehensive chain of clinical, pathological, and genetic evidence.

**TABLE 2:** Thalassemia-associated variants included in the genotyping assay.

Disease	Variant Type	Details
α-Thalassemia	16 deletions	--SEA, -α^3.7^, -α^4.2^, -α^2.4^, -α^27.6^, --Thai, --FIL, --MED, --^20.5^, HS-40 deletion, -α^21.9^, -αMAL^3.5^, -α^2.8^,--^11.1^, --^9.7^, other large fragment deletions
	4 homologous recombinations	ααα^anti3.7^, ααα^anti4.2^, HKαα, ααα^antiHKαα^
	1 HBA1 point mutations	c.223G>C
	9 HBA2 point mutations	c.40G>T, c.91_93delGAG, c.95G>A, c.99G>A, c.358C>T (Hb Constant Spring), c.369C>G, c.377T>C, c.427T>C, c.*92A>G
β-Thalassemia	8 deletions	Chinese (Aγδβ)0-deletion, HPFH-6 deletion, HPFH-S.E. Asian deletion, Thai (δβ)0-Thal deletion, Filipino deletion, Taiwanese deletion, Lepore-Boston-Washington deletion, other large fragment deletions
	37 HBB point mutations	c.-140C>T, c.-100G>A, c.-82C>A, c.-81A>G, c.-81A>C, c.-80T>C, c.-79A>G, c.-78A>G, c.-78A>C, c.-50A>C, c.-11_-8delAAAC, c.2T>G, c.17_18delCT, c.25_26delAA, c.27dup, c.45dupG, c.52A>T, c.79G>A, c.85dupC, c.91A>G, c.92 + 1G>T, c.92 + 2T>C, c.92 + 5G>C, c.92 + 6T>C, c.93-21G>A, c.94delC, c.113G>A, c.126_129delCTTT, c.130G>T, c.162delT, c.165_177del13, c.216dupT, c.217dupA, c.315 + 1G>A, c.315 + 5G>C, c.316–197C>T, c.383_385delAGG

Currently, there is no specific treatment for *LCAT* deficiency. Upon admission, the patient received symptomatic and supportive care to manage edema and slow the progression of chronic kidney disease, including the use of angiotensin-converting enzyme inhibitors and diuretics. After treatment, edema improved. Post-discharge, renal function, proteinuria, and lipid levels were closely monitored. After 3 months of follow-up, the patient had lost 5 kg. Laboratory tests showed the following results: hemoglobin 89 g/L, MCV 66.2 fL, MCH 21.2 pg, MCHC 320 g/L; renal function revealed urea nitrogen 14.83 mmol/L, creatinine 168.6 μmol/L, eGFR (CKD-EPI) 40.04 mL/min/1.73 m^2^; the lipid profile indicated triglyceride 0.72 mmol/L, total cholesterol 2.12 mmol/L, high-density lipoprotein cholesterol 0.11 mmol/L, low-density lipoprotein cholesterol 0.87 mmol/L (Table 3). These findings suggest a decline in renal function over this short follow-up period, although longer observation is needed to confirm this trajectory. Long-term follow-up will continue, and genetic counseling will be provided for the patient and their family, covering disease inheritance, carrier risk assessment, and fertility-related genetic counseling.

**TABLE 3:** Laboratory parameters at baseline and 3 months follow-up.

Parameter	Admission	3 months
Hemoglobin (g/L)	94	89
MCV (fL)	68.6	66.2
MCH (pg)	21.4	21.2
Urea nitrogen (mmol/L)	5.0	14.83
Serum creatinine (μmol/L)	115.5	168.6
eGFR (mL/min/1.73 m^2^)	63.25	40.04
High-density lipoprotein cholesterol (mmol/L)	0.43	0.11
Low-density lipoprotein cholesterol (mmol/L)	1.69	0.87
Total cholesterol (mmol/L)	3.85	2.12
Triglycerides (mmol/L)	1.73	0.72

## 3 Discussion

This is the first reported case of co-inherited *LCAT* deficiency and α^0^-thalassemia, confirmed by renal biopsy and comprehensive genetic testing. A homozygous *LCAT* mutation (c.355G>C, p.Gly119Arg) was identified, and expanded testing revealed α^0^-thalassemia trait (--SEA/αα). This case underscores the diagnostic challenge when multi-system involvement cannot be explained by a single disease.

*LCAT* deficiency is a rare autosomal recessive disorder caused by the dysfunction of the LCAT enzyme, leading to impaired cholesterol esterification and subsequent lipoprotein metabolism abnormalities (Strazzella et al., 2021). Clinically, the disease is typically characterized by significant reductions in HDL-C, corneal opacity, and progressive renal damage. Some patients may also present with hemolytic anemia (Najafian et al., 2017). In this case, the patient showed significantly low HDL-C and nephrotic syndrome after admission. Combined with the presence of corneal opacity since childhood, the clinical suspicion of hereditary lipoprotein metabolic abnormalities related nephropathy was strongly suspected. Renal biopsy revealed characteristic glomerular lipid deposition, providing the pathological evidence needed to confirm the diagnosis. The homozygous *LCAT* gene mutation, along with family verification (parents as heterozygous carriers), formed a complete clinical-pathological-genetic evidence chain.

An important diagnostic consideration in this case is the patient’s anemia, which presented as microcytic hypochromic anemia. This did not fully match the typical anemia phenotype seen in *LCAT* deficiency. Normal ferritin excluded iron deficiency, and increased osmotic fragility suggested an inherited RBC disorder. Given parental consanguinity, we expanded testing and identified α^0^-thalassemia. This finding is clinically significant—it explains the anemia and prevents unnecessary iron supplementation, which risks iron overload in thalassemia (Lal and Vichinsky, 2023; Musallam et al., 2024).

From a genetic perspective, *LCAT* (16q22.1) and the *HBA* (α-globin) cluster (16p13.3) are ∼25 Mb apart on chromosome 16 and typically inherited independently. However, in consanguineous families, shared haplotypes increase the likelihood of homozygosity for multiple recessive alleles on the same chromosome (Hildebrandt et al., 2009). This patient’s co-inherited disorders likely reflect this mechanism. As a single observation, however, this cannot establish causality but highlights the need to consider multiple recessive diagnoses in consanguineous families.

No specific treatment exists for *LCAT* deficiency. Management focuses on controlling edema and delaying CKD progression with ACE inhibitors, ARBs, and diuretics (Miarka et al., 2011; Vitali et al., 2022; Vitali et al., 2023). Conventional lipid-lowering therapies are ineffective, as the primary defect is enzyme inactivation, not lipid overproduction. *LCAT* dysfunction impairs HDL maturation and reverse cholesterol transport, leading to accumulation of lipoprotein-X (Lp-X) particles (Ossoli et al., 2021; Pavanello et al., 2022). These particles deposit in the glomerular basement membrane, triggering inflammation and fibrosis independent of circulating lipid levels (Pavanello et al., 2020), and may also account for the rapid deterioration observed in this patient. Indeed, renal biopsy revealed extensive Lp-X deposition and foot process fusion (Figure 5), indicating advanced glomerular damage at diagnosis that likely reflected long-standing Lp-X-induced injury. Over the 3-month follow-up, eGFR declined from 63 to 40 mL/min/1.73 m^2^—a rate exceeding the annual decline of 3–5 mL/min/1.73 m^2^ reported in *LCAT* deficiency cohorts (Pavanello et al., 2020; Vitali et al., 2022). Contributing factors may include the absence of prior renoprotective therapy and the possibility that the disease had already reached an advanced stage with accelerated progression. Nevertheless, longer follow-up is required to determine whether this trajectory represents sustained rapid progression. Future therapies may target LCAT enzyme reactivation; small-molecule allosteric activators have shown promise *in vitro* for specific mutations (Vitali et al., 2023; Manthei et al., 2024). For patients with concurrent α^0^-thalassemia, regular monitoring of anemia and iron status is advised to prevent inappropriate iron supplementation.

There are still some limitations in this case. As this is a single case, the findings are hypothesis-generating. The follow-up period is short, and LCAT enzyme activity could not be measured because the test is unavailable in our region. Nonetheless, the diagnosis is robustly supported by clinical, pathological, and genetic evidence, including consistent *in silico* predictions (PolyPhen-2: 0.998, SIFT: 0.00, CADD: 28.5). Future studies with longer follow-up and multi-center case accumulation will help clarify the natural history and optimal management of co-inherited genetic conditions.

In conclusion, to the best of our knowledge, this case represents the first documented co-inheritance of *LCAT* deficiency and α^0^-thalassemia, supported by comprehensive clinical, pathological, and genetic evidence. It highlights the need for vigilance in diagnosing recessive genetic diseases, particularly in patients with complex phenotypes or in consanguineous families. Accurate diagnosis, supported by renal pathology and extensive genetic testing, is essential to guide personalized treatment and genetic counseling.

## Data Availability

The datasets presented in this study can be found in online repositories. The names of the repository/repositories and accession number(s) can be found in the article/Supplementary Material.
