# Supplementary material for: Case report: co-inheritance of familial lecithin-cholesterol acyltransferase deficiency and α0-Thalassemia
Source: Front Genet. 2026 Apr 17;17:1806855. doi: 10.3389/fgene.2026.1806855 (PMC13132501; doi:10.3389/fgene.2026.1806855)
Supplement: Supplementary file 1 [file DataSheet1.pdf]

Supplementary Material

1 Supplementary Figures

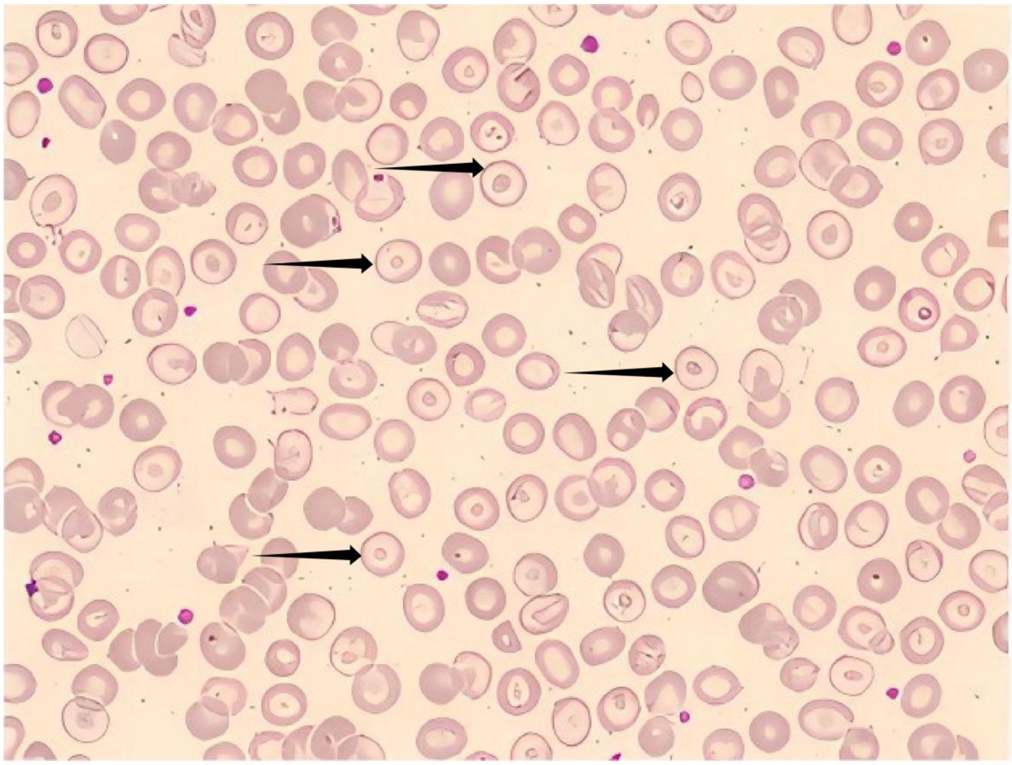

Figure 1. Peripheral blood smear (Wright-Giemsa stain) showing uneven red blood cell size, small cell hypochromasia, and numerous target cells (black arrows). Original magnification ×1000.

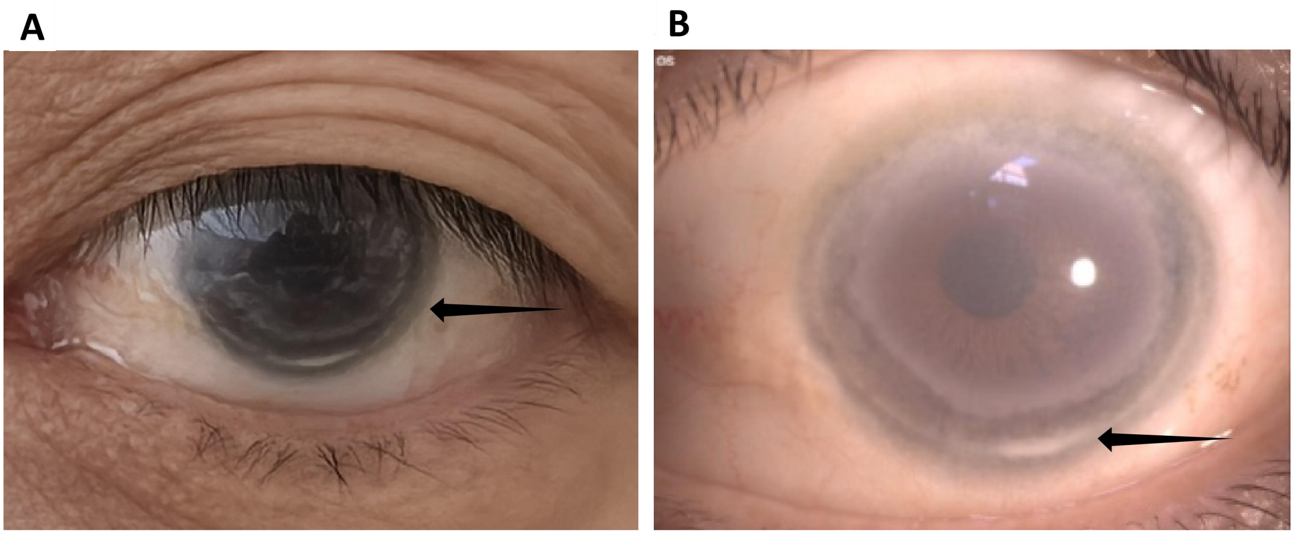

Figure 2. (A) The patient's cornea was cloudy, exhibiting fisheye changes. (B) Slit-lamp examination showing an annular turbid zone (black arrow) around the cornea. (Clinical images, no magnificat)

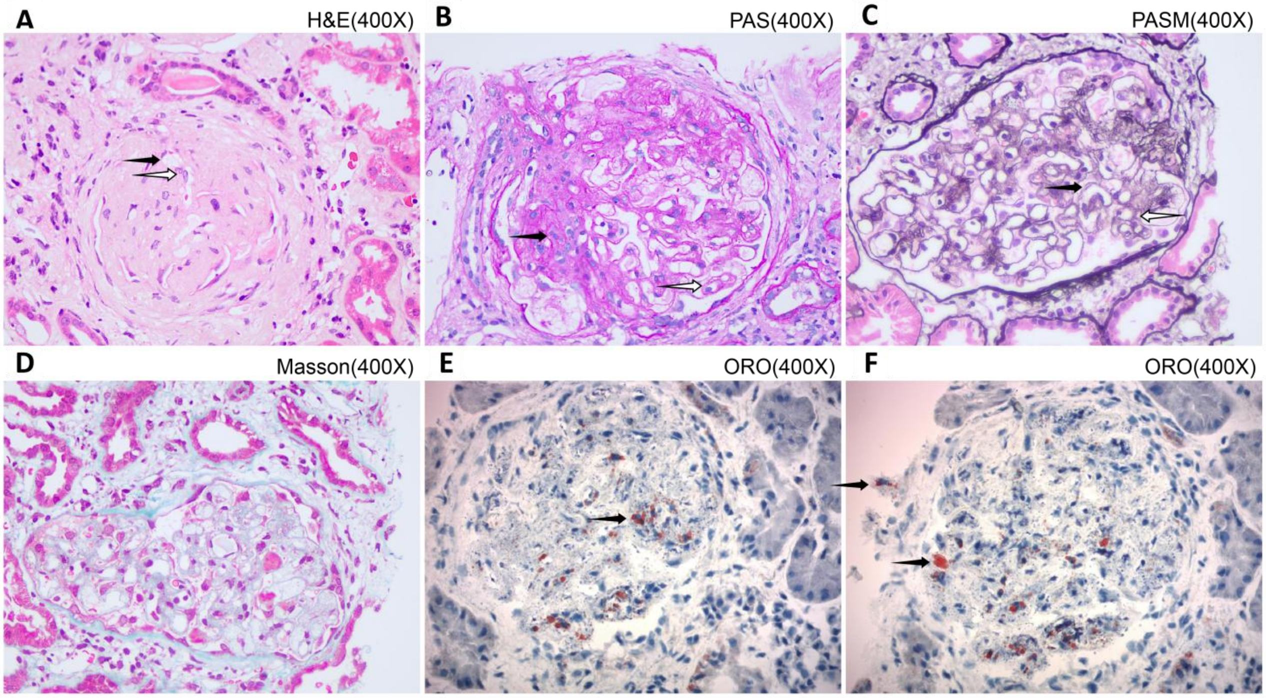

Figure 3. Light microscopy findings on renal biopsy. (A) Hematoxylin-eosin (H&E) staining showing highly dilated glomerular capillary loops, with the lumen filled by a lightly stained vacuolar thrombus-like substance (black arrow) and foamy degeneration of the endothelial cells (white arrow). (B) Periodic acid-Schiff (PAS) staining revealing mild to moderate diffuse proliferation of mesangial cells and matrix (black arrow), along with diffuse thickening of the glomerular basement membrane (white arrow). (C) Silver staining (PASM) highlighting vacuolar transparent areas of various sizes in the thickened basement membrane (white arrow), with a double-track sign caused by segmental mesangial matrix insertion (black arrow). (D) Masson's staining showing no Fuchsin deposition in

the mesangial, subepithelial, or subendothelial areas, ruling out common immune complex-mediated glomerulonephritis. (E-F) Oil red O staining demonstrating positive reabsorption granules in glomeruli and a few renal tubular epithelial cells (black arrows), confirming the presence of neutral lipid components. Original magnification ×400 for all panels.

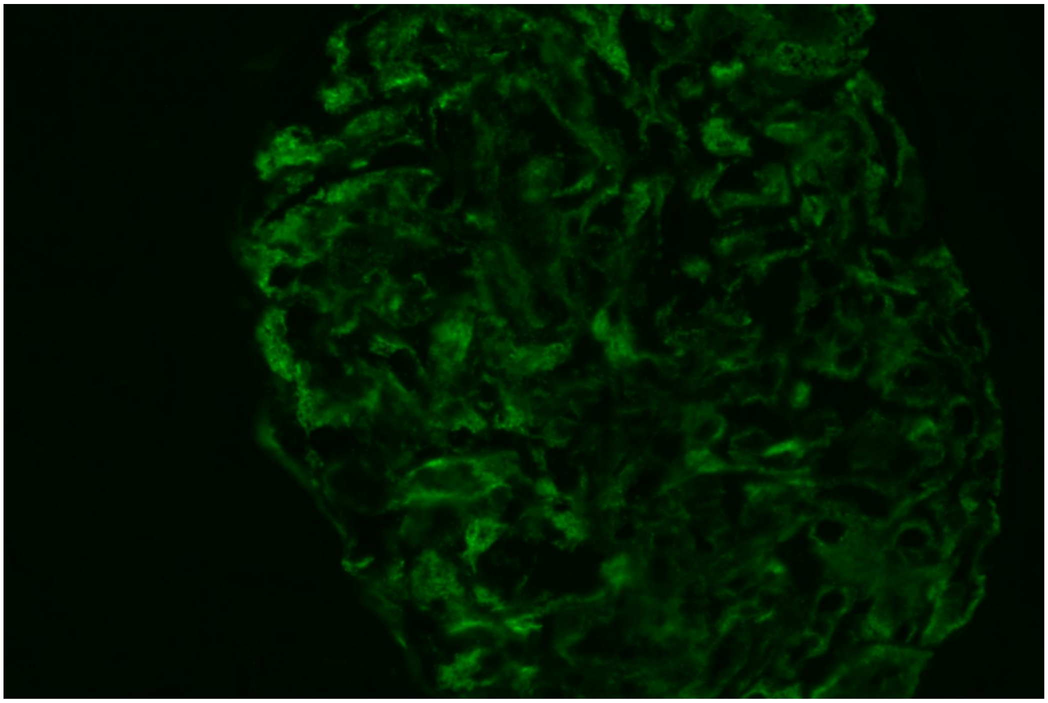

APOB(400X)

Figure 4. Immunofluorescence staining showing ApoB+, with diffuse, granular deposition primarily in the lumina of the glomerular capillary loops and the mesangial areas. Original magnification ×400.

In addition, IgA, IgG, IgM, C3, C4, C1q, fibrinogen, κ and λ light chains were all negative (images not shown).

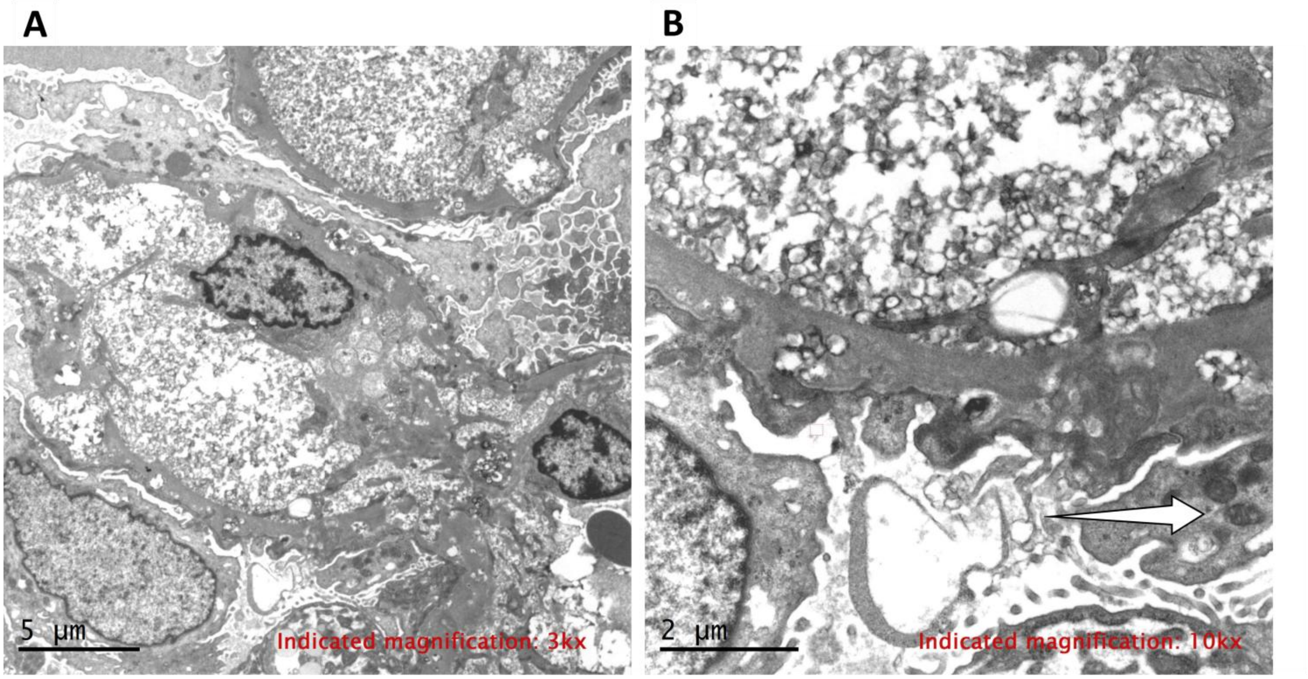

Figure 5. Electron microscopy findings on renal biopsy (uranyl acetate and lead citrate stain). (A) Extensive lipid accumulation in the glomerular basement membrane, mesangial matrix, and endothelial cells with diffuse foot process fusion. (B) Some vacuoles containing osmiophilic lamellar structures (white arrow). Original magnification ×3000 in A and ×10000 in B.

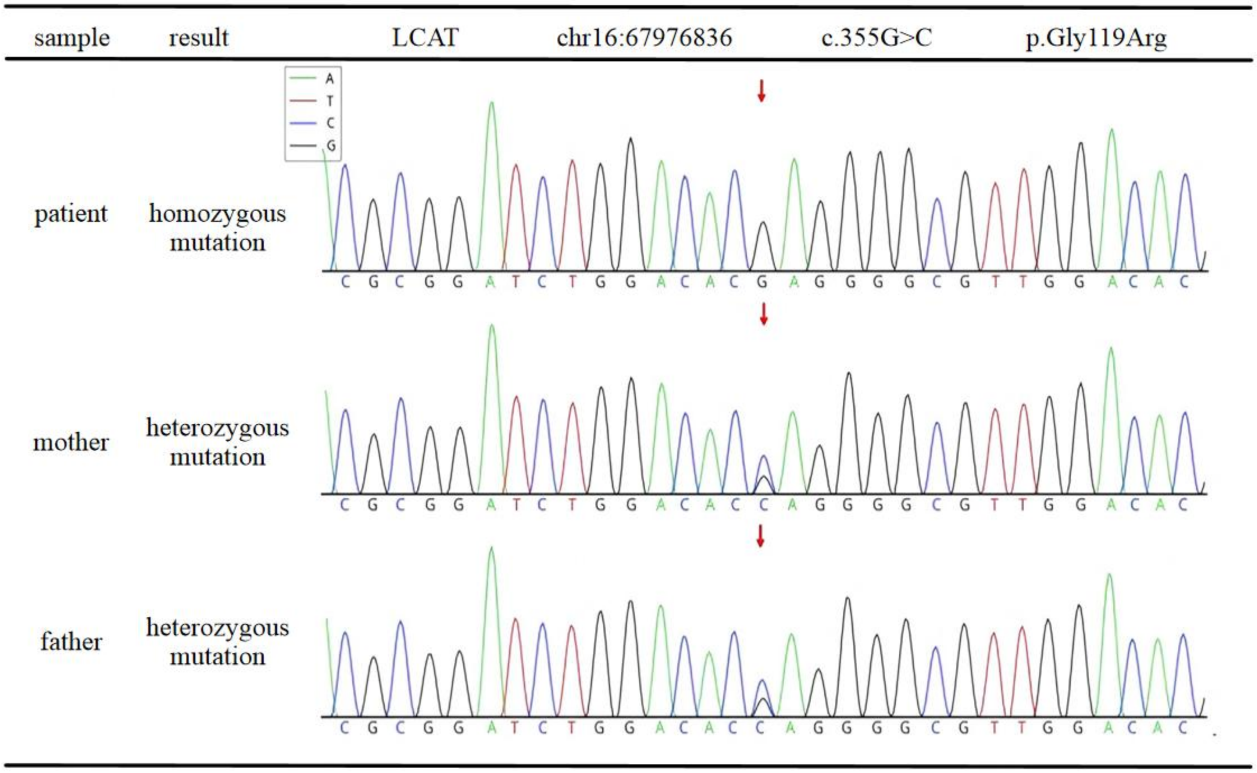

Figure 6. *LCAT* gene analysis and family verification: The patient was homozygous for the c.355G>C variant. Both parents were asymptomatic carriers of the heterozygous c.355G>C variant.

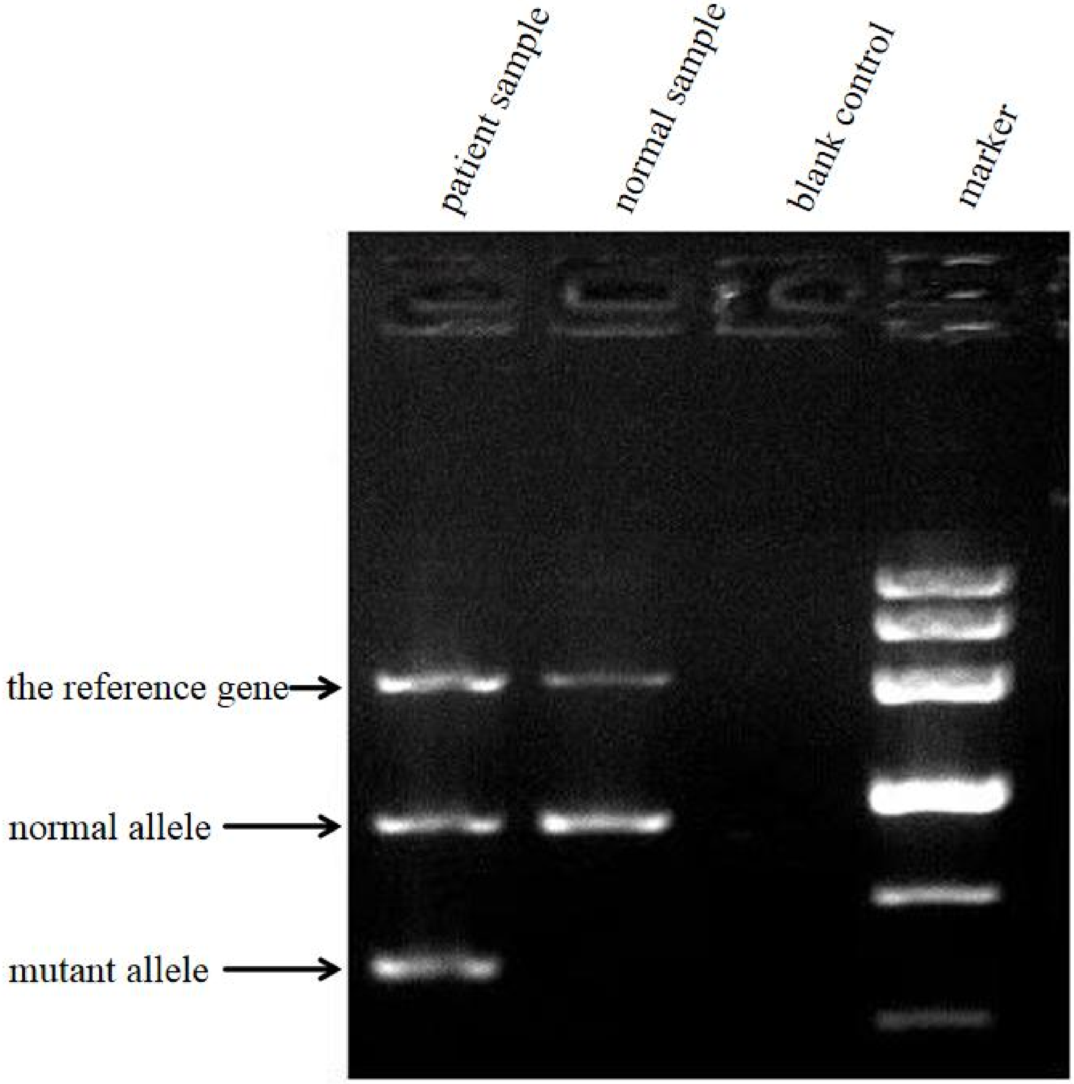

Figure 7. Results of agarose gel electrophoresis of thalassemia-related genes in α^0-thalassemia.

Table 1. In silico pathogenicity predictions for the *LCAT* c.355G>C (p.Gly119Arg) variant.

Prediction tool	Website	Score	Threshold	Prediction
PolyPhen-2	http://genetics.bwh.harvard.edu/pph2/	0.998	>0.9 = probably damaging	Probably damaging
SIFT	https://sift.bii.a-star.edu.sg/	0.00	≤0.05 = deleterious	Deleterious
CADD	https://cadd.gs.washington.edu/	28.5	>20 = clinically significant	Clinically significant

Table 2. Thalassemia-Associated Variants Included in the Genotyping Assay

Disease	Variant Type	Details
α-Thalassemia	16 Deletions	--SEA, -$\alpha^{3.7}$, -$\alpha^{4.2}$, -$\alpha^{2.4}$, -$\alpha^{27.6}$, --THAI, --FIL, --MED, --$^{20.5}$, HS-40 deletion, -$\alpha^{21.9}$, -αMAL$^{3.5}$, -$\alpha^{2.8}$,--$^{11.1}$, --$^{9.7}$, other large fragment deletions
	4 Homologous recombinations	$\alpha\alpha\alpha^{anti3.7}$, $\alpha\alpha\alpha^{anti4.2}$, HKαα, $\alpha\alpha\alpha^{antiHK\alpha\alpha}$
	1 *HBA1* point mutations	c.223G>C
	9 *HBA2* point mutations	c.40G>T, c.91_93delGAG, c.95G>A, c.99G>A, c.358C>T (Hb Constant Spring), c.369C>G, c.377T>C, c.427T>C, c.*92A>G
β-Thalassemia	8 Deletions	Chinese (Aγδβ)0-deletion, HPFH-6 deletion, HPFH-S.E. Asian deletion, Thai (δβ)0-Thal deletion, Filipino deletion, Taiwanese deletion, Lepore-Boston-Washington deletion, other large fragment deletions
	37 *HBB* point mutations	c.-140C>T, c.-100G>A, c.-82C>A, c.-81A>G, c.-81A>C, c.-80T>C, c.-79A>G, c.-78A>G, c.-78A>C, c.-50A>C, c.-11_-8delAAAC, c.2T>G, c.17_18delCT, c.25_26delAA, c.27dup, c.45dupG, c.52A>T, c.79G>A, c.85dupC, c.91A>G, c.92+1G>T, c.92+2T>C, c.92+5G>C, c.92+6T>C, c.93-21G>A, c.94delC, c.113G>A, c.126_129delCTTT, c.130G>T, c.162delT, c.165_177del13, c.216dupT, c.217dupA, c.315+1G>A, c.315+5G>C, c.316-197C>T, c.383_385delAGG

Table 3. Laboratory parameters at baseline and 3 months follow-up

Parameter	Admission	3 months
Hemoglobin (g/L)	94	89
MCV (fL)	68.6	66.2
MCH (pg)	21.4	21.2
Urea nitrogen (mmol/L)	5.0	14.83
Serum Creatinine (μmol/L)	115.5	168.6
eGFR (mL/min/1.73 m^2)	63.25	40.04
High-density lipoprotein cholesterol (mmol/L)	0.43	0.11
Low-density lipoprotein cholesterol (mmol/L)	1.69	0.87
Total cholesterol (mmol/L)	3.85	2.12
Triglycerides (mmol/L)	1.73	0.72
